# (Tris{2-[(5-hy­droxy­pyridin-2-yl-κ*N*)methyl­idene­imino-κ*N*]eth­yl}amine)­zinc dinitrate

**DOI:** 10.1107/S1600536811048094

**Published:** 2011-11-19

**Authors:** Maksym Seredyuk, Kateryna O. Znovjyak, Matti Haukka, Vadim A. Pavlenko, Nadezhda A. Bokach

**Affiliations:** a National Taras Shevchenko University, Department of Chemistry, Volodymyrska Str. 64, 01033 Kyiv, Ukraine; b Department of Chemistry, University of Joensuu, PO Box 111, 80101 Joensuu, Finland; c Department of Chemistry, St. Petersburg State University, Universitetsky Pr. 26, 198504 Stary Petergof, Russian Federation

## Abstract

In the complex cation of the title compound, [Zn(C_24_H_27_N_7_O_3_)](NO_3_)_2_, the tripodal tris­{[2-(5-hy­droxy­pyridin-2-yl)methyl­idene­imino]­eth­yl}amine ligand is coordin­ated to the Zn atom through the three pyridine and three imino N atoms. The coordination sphere of the Zn atom is based on an octahedron with a significant distortion towards trigonal prismatic, the twist angle being 45.58 (9)°. The crystal packing is formed by *L* and *D* anti­podes arranged in layers disposed parallel to the *b* axis. Strong O—H⋯O hydrogen bonding exists between the hy­droxy groups of the ligand and the nitrate anion.

## Related literature

For structural and magnetic studies of related tripodand-based complexes of iron(II), see: Seredyuk *et al.* (2007 ▶, 2008 ▶, 2011 ▶). For related structures, see: Petrusenko *et al.* (1997 ▶); Krämer & Fritsky (2000 ▶); Świątek-Kozłowska *et al.* (2000 ▶); Wörl *et al.* (2005 ▶); Sachse *et al.* (2008 ▶); Moroz *et al.* (2010 ▶). 
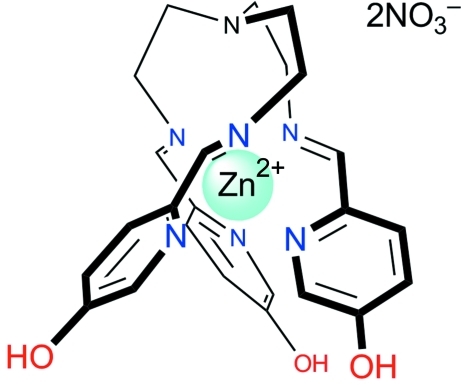


## Experimental

### 

#### Crystal data

[Zn(C_24_H_27_N_7_O_3_)](NO_3_)_2_
*M* *_r_* = 650.92
Monoclinic, 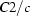

*a* = 28.0587 (12) Å
*b* = 10.3677 (2) Å
*c* = 19.1322 (8) Å
β = 101.277 (2)°
*V* = 5458.2 (3) Å^3^
*Z* = 8
Mo *K*α radiation
μ = 0.97 mm^−1^
*T* = 120 K
0.30 × 0.23 × 0.12 mm

#### Data collection

Nonius KappaCCD diffractometer
Absorption correction: multi-scan (*DENZO*/*SCALEPACK*; Otwinowski & Minor, 1997 ▶) *T*_min_ = 0.764, *T*_max_ = 0.871
13185 measured reflections
4754 independent reflections
3823 reflections with *I* > 2σ(*I*)
*R*_int_ = 0.024

#### Refinement

*R*[*F* ^2^ > 2σ(*F* ^2^)] = 0.029
*wR*(*F* ^2^) = 0.064
*S* = 1.05
4754 reflections
400 parameters
H atoms treated by a mixture of independent and constrained refinement
Δρ_max_ = 0.48 e Å^−3^
Δρ_min_ = −0.39 e Å^−3^

### 

Data collection: *COLLECT* (Bruker–Nonius, 2000 ▶); cell refinement: *DENZO*/*SCALEPACK* (Otwinowski & Minor, 1997 ▶); data reduction: *DENZO*/*SCALEPACK*; program(s) used to solve structure: *SIR2004* (Burla *et al.*, 2003 ▶); program(s) used to refine structure: *SHELXL97* (Sheldrick, 2008 ▶); molecular graphics: *DIAMOND* (Brandenburg, 2011 ▶); software used to prepare material for publication: *SHELXL97*.

## Supplementary Material

Crystal structure: contains datablock(s) I, global. DOI: 10.1107/S1600536811048094/yk2030sup1.cif

Structure factors: contains datablock(s) I. DOI: 10.1107/S1600536811048094/yk2030Isup2.hkl

Supplementary material file. DOI: 10.1107/S1600536811048094/yk2030Isup3.cdx

Additional supplementary materials:  crystallographic information; 3D view; checkCIF report

## Figures and Tables

**Table 1:** Hydrogen-bond geometry (Å, °)

*D*—H⋯*A*	*D*—H	H⋯*A*	*D*⋯*A*	*D*—H⋯*A*
O3—H3*o*⋯O6	0.80 (3)	1.90 (3)	2.698 (2)	173 (3)
O1—H1*o*⋯O7	0.82 (3)	1.80 (3)	2.597 (2)	163 (3)
O2—H2*o*⋯O4^i^	0.78 (3)	1.84 (3)	2.593 (3)	162 (3)

Symmetry code: (i) 
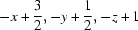
.

**Fig. 1.**
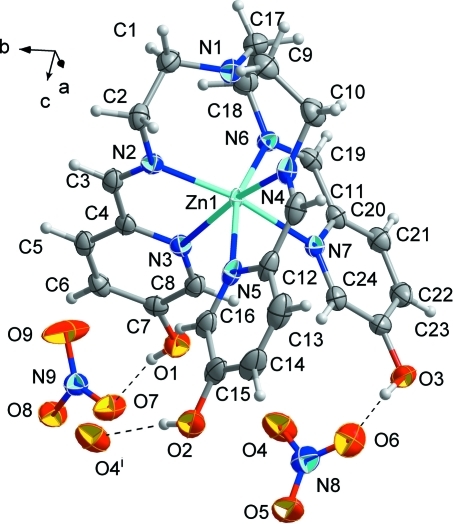
The title molecule with displacement ellipsoids drawn at the 50% probability level. Dashed lines show hydrogen bonds between complex cations and nitrate anions [symmetry code: (i) 1.5–x, 0.5–y, 1–z].

**Fig. 2.**
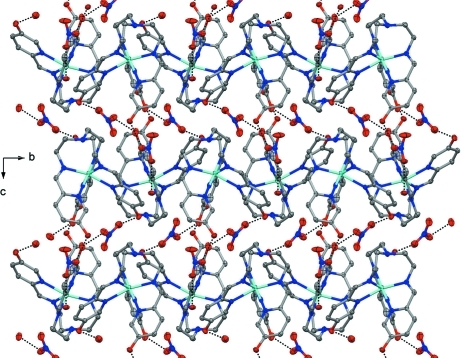
Projection of the molecular packing along the a axis. Dashed lines correspond to hydrogen bonds between complex cations and nitrate anions.

## supplementary crystallographic information

### Comment

As a part of our study of the tripodand-based 3*d*-metal complexes (Seredyuk *et al.*, 2007; Seredyuk *et al.*, 2008; Seredyuk *et al.*, 2011), we report the crystal structure of the title compound.

The tripod ligand tris[2-(5-hydroxy-2-pyridylmethyleneimino)ethyl]amine coordinates to the metal centre through the three pyridine and three imino N atoms (Fig. 1). The coordination polyhedron of the zinc ion in the complex molecule is a distorted trigonal antiprism with the twist angle equal to 46.71 (8)°. The tertiary capping N1 atom lies on the pseudo *C*_3_–axis of the molecule and is situated at 2.972 (2) Å from the Zn center. The average values for the Zn–N*^py^* and the Zn–N*^im^* bond lengths differ significantly and are 2.265 (2) and 2.132 (2) Å, respectively. Similar differences in the geometrical parameters have been observed before in the related zinc complexes (Petrusenko *et al.*,1997; Świątek-Kozłowska *et al.*, 2000; Wörl *et al.*, 2005). The C—C and C—N bond lengths in the pyridine rings are normal for 2-substituted pyridine derivatives (Krämer & Fritsky, 2000; Sachse *et al.*, 2008; Moroz *et al.*, 2010).

The crystal packing is formed by *L* and *D* antipodes arranged in layers disposed parallel to the *b* axis (Fig. 2). A strong hydrogen bonding is settled between hydroxy groups of the ligand and nitrate anions [2.593 (3)–2.698 (3) Å].

### Experimental

To a stirred boiling mixture of 5-hydroxy-picolinaldehyde (Seredyuk *et al.*, 2008) (0.5 g, 0.406 mmol) and Zn(NO_3_)_2_.6H_2_O (0.403 g, 0.136 mmol) in ethanol was added tris(2-aminoethyl)amine (0.198 g, 0.136 mmol). The obtained mixture was kept boiling for 15 min and then transferred into a fridge and left at 4°C overnight. The crystalline precipitate was filtered off, washed with a small portion of cold ethanol and air dried. Recrystallization in a thermostat from boiling methanol provided colorless crystalline material. ESI MS (*rel*. int.): *m*/*z* 587 [*M*+NO_3_]^+^ (17%), 524 [M–H]^+^ (100%) 264 [*M*]^++^ (18%). Calc for C_24_H_27_N_9_O_9_Zn: C, 44.28, H, 4.18, N, 19.37. Found C, 44.20, H, 4.28, N, 19.27.

### Refinement

The OH hydrogen atoms were located from the difference Fourier map, and their positional and thermal parameters were refined freely. The CH hydrogen atoms were positioned geometrically and refined as riding atoms, with C–H = 0.95–0.99 Å and with *U*_iso_ = 1.2 *U*_eq_(parent atom).

### Crystal data

**Table:**

[Zn(C_24_H_27_N_7_O_3_)](NO_3_)_2_	*F*(000) = 2688
*M**_r_* = 650.92	*D*_x_ = 1.584 Mg m^−^^3^
Monoclinic, *C*2/*c*	Mo *K*α radiation, λ = 0.71073 Å
Hall symbol: -C 2yc	Cell parameters from 3966 reflections
*a* = 28.0587 (12) Å	θ = 1.5–25.5°
*b* = 10.3677 (2) Å	µ = 0.97 mm^−^^1^
*c* = 19.1322 (8) Å	*T* = 120 K
β = 101.277 (2)°	Block, colorless
*V* = 5458.2 (3) Å^3^	0.30 × 0.23 × 0.12 mm
*Z* = 8	

### Data collection

**Table:**

Nonius KappaCCD diffractometer	4754 independent reflections
Radiation source: fine-focus sealed tube	3823 reflections with *I* > 2σ(*I*)
horizontally mounted graphite crystal	*R*_int_ = 0.024
Detector resolution: 9 pixels mm^-1^	θ_max_ = 25.0°, θ_min_ = 2.9°
φ scans and ω scans with κ offset	*h* = −27→33
Absorption correction: multi-scan (*DENZO*/*SCALEPACK*; Otwinowski & Minor, 1997)	*k* = −12→10
*T*_min_ = 0.764, *T*_max_ = 0.871	*l* = −22→20
13185 measured reflections	

### Refinement

**Table:**

Refinement on *F*^2^	Primary atom site location: structure-invariant direct methods
Least-squares matrix: full	Secondary atom site location: difference Fourier map
*R*[*F*^2^ > 2σ(*F*^2^)] = 0.029	Hydrogen site location: inferred from neighbouring sites
*wR*(*F*^2^) = 0.064	H atoms treated by a mixture of independent and constrained refinement
*S* = 1.05	*w* = 1/[σ^2^(*F*_o_^2^) + (0.0217*P*)^2^ + 8.7493*P*] where *P* = (*F*_o_^2^ + 2*F*_c_^2^)/3
4754 reflections	(Δ/σ)_max_ = 0.001
400 parameters	Δρ_max_ = 0.48 e Å^−^^3^
0 restraints	Δρ_min_ = −0.39 e Å^−^^3^

### Special details

**Table:**

Experimental. The OH hydrogen atoms were located from the difference Fourier map, and their positional and thermal parameters were refined freely. The CH hydrogen atoms were positioned geometrically and refined as riding atoms, with C—H = 0.95 - 0.99 Å and with Uĩso~ = 1.2 U~eq~(parent atom).
Geometry. All e.s.d.'s (except the e.s.d. in the dihedral angle between two l.s. planes) are estimated using the full covariance matrix. The cell e.s.d.'s are taken into account individually in the estimation of e.s.d.'s in distances, angles and torsion angles; correlations between e.s.d.'s in cell parameters are only used when they are defined by crystal symmetry. An approximate (isotropic) treatment of cell e.s.d.'s is used for estimating e.s.d.'s involving l.s. planes.
Refinement. Refinement of *F*^2^ against ALL reflections. The weighted *R*-factor *wR* and goodness of fit *S* are based on *F*^2^, conventional *R*-factors *R* are based on *F*, with *F* set to zero for negative *F*^2^. The threshold expression of *F*^2^ > σ(*F*^2^) is used only for calculating *R*-factors(gt) *etc*. and is not relevant to the choice of reflections for refinement. *R*-factors based on *F*^2^ are statistically about twice as large as those based on *F*, and *R*- factors based on ALL data will be even larger.

### Fractional atomic coordinates and isotropic or equivalent isotropic displacement parameters (Å^2^)

**Table:**

	*x*	*y*	*z*	*U*_iso_*/*U*_eq_	
Zn1	0.634474 (9)	0.16452 (2)	0.235644 (13)	0.01525 (8)	
O1	0.58450 (6)	0.16550 (16)	0.50553 (8)	0.0220 (4)	
O2	0.79699 (7)	0.28797 (17)	0.44693 (11)	0.0340 (5)	
O3	0.72303 (6)	−0.23705 (16)	0.42204 (9)	0.0266 (4)	
O4	0.74429 (6)	0.03487 (17)	0.48994 (9)	0.0332 (4)	
O5	0.81700 (7)	0.03610 (17)	0.55434 (10)	0.0438 (5)	
O6	0.80039 (6)	−0.07908 (17)	0.45764 (9)	0.0322 (4)	
O7	0.56606 (5)	0.26383 (16)	0.62224 (8)	0.0268 (4)	
O8	0.51315 (6)	0.33927 (16)	0.68067 (8)	0.0258 (4)	
O9	0.49886 (7)	0.3452 (2)	0.56617 (9)	0.0595 (7)	
N1	0.58398 (6)	0.20629 (17)	0.08571 (9)	0.0185 (4)	
N2	0.61171 (6)	0.35640 (16)	0.20881 (9)	0.0151 (4)	
N3	0.60926 (6)	0.23962 (16)	0.33387 (9)	0.0158 (4)	
N4	0.67412 (7)	0.10515 (17)	0.15702 (10)	0.0200 (4)	
N5	0.71145 (6)	0.20475 (16)	0.28566 (10)	0.0180 (4)	
N6	0.56701 (6)	0.06303 (16)	0.20400 (9)	0.0156 (4)	
N7	0.64650 (6)	−0.03002 (16)	0.29422 (9)	0.0149 (4)	
N8	0.78803 (7)	−0.00173 (19)	0.50157 (11)	0.0266 (5)	
N9	0.52514 (6)	0.31521 (17)	0.62315 (9)	0.0190 (4)	
C1	0.57457 (8)	0.3445 (2)	0.08296 (11)	0.0212 (5)	
H1A	0.5415	0.3611	0.0916	0.025*	
H1B	0.5763	0.3777	0.0350	0.025*	
C2	0.61165 (8)	0.4145 (2)	0.13889 (11)	0.0196 (5)	
H2A	0.6444	0.4072	0.1271	0.024*	
H2B	0.6032	0.5071	0.1397	0.024*	
C3	0.59253 (7)	0.4176 (2)	0.25399 (11)	0.0162 (5)	
H3	0.5801	0.5020	0.2431	0.019*	
C4	0.58951 (7)	0.35904 (19)	0.32235 (11)	0.0149 (5)	
C5	0.56781 (7)	0.4207 (2)	0.37240 (11)	0.0171 (5)	
H5	0.5545	0.5046	0.3630	0.020*	
C6	0.56555 (8)	0.3599 (2)	0.43585 (11)	0.0176 (5)	
H6	0.5507	0.4007	0.4706	0.021*	
C7	0.58551 (7)	0.2375 (2)	0.44754 (11)	0.0158 (5)	
C8	0.60739 (7)	0.1816 (2)	0.39515 (11)	0.0167 (5)	
H8	0.6215	0.0985	0.4038	0.020*	
C9	0.62025 (8)	0.1637 (2)	0.04540 (12)	0.0239 (5)	
H9A	0.6407	0.2379	0.0372	0.029*	
H9B	0.6037	0.1303	−0.0016	0.029*	
C10	0.65213 (9)	0.0588 (2)	0.08562 (12)	0.0254 (5)	
H10A	0.6323	−0.0189	0.0895	0.031*	
H10B	0.6780	0.0351	0.0594	0.031*	
C11	0.72019 (8)	0.1053 (2)	0.17658 (12)	0.0229 (5)	
H11	0.7400	0.0734	0.1455	0.028*	
C12	0.74247 (8)	0.1546 (2)	0.24709 (13)	0.0210 (5)	
C13	0.79224 (8)	0.1498 (2)	0.27344 (14)	0.0298 (6)	
H13	0.8134	0.1144	0.2453	0.036*	
C14	0.81062 (8)	0.1967 (2)	0.34045 (15)	0.0307 (6)	
H14	0.8446	0.1947	0.3590	0.037*	
C15	0.77898 (8)	0.2468 (2)	0.38054 (13)	0.0248 (6)	
C16	0.72922 (8)	0.2496 (2)	0.35089 (12)	0.0204 (5)	
H16	0.7074	0.2846	0.3781	0.024*	
C17	0.54127 (8)	0.1244 (2)	0.07905 (11)	0.0207 (5)	
H17A	0.5486	0.0382	0.0615	0.025*	
H17B	0.5143	0.1624	0.0440	0.025*	
C18	0.52591 (8)	0.1106 (2)	0.15087 (12)	0.0205 (5)	
H18A	0.5151	0.1953	0.1661	0.025*	
H18B	0.4984	0.0496	0.1466	0.025*	
C19	0.56437 (8)	−0.0477 (2)	0.23137 (11)	0.0162 (5)	
H19	0.5354	−0.0968	0.2184	0.019*	
C20	0.60524 (7)	−0.1008 (2)	0.28262 (11)	0.0153 (5)	
C21	0.60142 (8)	−0.2162 (2)	0.31789 (11)	0.0196 (5)	
H21	0.5719	−0.2636	0.3089	0.024*	
C22	0.64107 (8)	−0.2615 (2)	0.36629 (12)	0.0219 (5)	
H22	0.6391	−0.3396	0.3915	0.026*	
C23	0.68378 (8)	−0.1907 (2)	0.37740 (11)	0.0189 (5)	
C24	0.68483 (8)	−0.0745 (2)	0.34015 (11)	0.0172 (5)	
H24	0.7140	−0.0256	0.3480	0.021*	
H1O	0.5743 (11)	0.204 (3)	0.5368 (16)	0.052 (10)*	
H3O	0.7447 (11)	−0.185 (3)	0.4311 (15)	0.046 (9)*	
H2O	0.7795 (11)	0.332 (3)	0.4639 (15)	0.041 (10)*	

### Atomic displacement parameters (Å^2^)

**Table:**

	*U*^11^	*U*^22^	*U*^33^	*U*^12^	*U*^13^	*U*^23^
Zn1	0.01257 (13)	0.01396 (13)	0.01942 (14)	0.00079 (10)	0.00361 (10)	−0.00027 (11)
O1	0.0257 (9)	0.0234 (9)	0.0185 (9)	0.0036 (7)	0.0079 (7)	0.0033 (7)
O2	0.0229 (10)	0.0248 (10)	0.0474 (12)	0.0004 (8)	−0.0096 (9)	−0.0060 (9)
O3	0.0275 (10)	0.0210 (9)	0.0264 (9)	0.0007 (8)	−0.0067 (8)	0.0040 (7)
O4	0.0195 (9)	0.0450 (11)	0.0349 (10)	−0.0054 (8)	0.0052 (8)	−0.0108 (8)
O5	0.0396 (11)	0.0296 (10)	0.0500 (12)	−0.0054 (8)	−0.0213 (10)	−0.0024 (9)
O6	0.0293 (10)	0.0373 (10)	0.0331 (10)	0.0027 (8)	0.0136 (8)	0.0017 (8)
O7	0.0161 (9)	0.0407 (10)	0.0246 (9)	0.0085 (7)	0.0062 (7)	0.0018 (7)
O8	0.0283 (9)	0.0355 (10)	0.0152 (8)	0.0071 (8)	0.0081 (7)	−0.0018 (7)
O9	0.0454 (12)	0.1089 (19)	0.0204 (10)	0.0417 (13)	−0.0030 (9)	−0.0021 (11)
N1	0.0197 (10)	0.0170 (9)	0.0189 (10)	0.0016 (8)	0.0037 (8)	−0.0005 (8)
N2	0.0136 (9)	0.0152 (9)	0.0160 (9)	−0.0010 (7)	0.0019 (8)	−0.0004 (7)
N3	0.0134 (9)	0.0156 (9)	0.0179 (10)	0.0000 (7)	0.0019 (8)	−0.0016 (8)
N4	0.0205 (11)	0.0156 (9)	0.0253 (11)	0.0023 (8)	0.0080 (9)	0.0014 (8)
N5	0.0144 (10)	0.0125 (9)	0.0276 (11)	0.0006 (7)	0.0051 (8)	0.0046 (8)
N6	0.0141 (9)	0.0164 (9)	0.0164 (10)	0.0013 (7)	0.0029 (8)	−0.0013 (8)
N7	0.0164 (10)	0.0132 (9)	0.0158 (9)	0.0000 (7)	0.0049 (8)	−0.0026 (7)
N8	0.0264 (12)	0.0262 (11)	0.0271 (12)	−0.0070 (9)	0.0048 (10)	0.0067 (9)
N9	0.0194 (10)	0.0203 (10)	0.0162 (10)	−0.0005 (8)	0.0009 (8)	−0.0002 (8)
C1	0.0254 (12)	0.0208 (12)	0.0169 (11)	0.0041 (10)	0.0027 (10)	0.0038 (10)
C2	0.0238 (12)	0.0172 (12)	0.0191 (12)	0.0024 (9)	0.0076 (10)	0.0021 (9)
C3	0.0135 (11)	0.0123 (11)	0.0221 (12)	−0.0002 (9)	0.0017 (9)	−0.0003 (9)
C4	0.0109 (11)	0.0140 (11)	0.0197 (12)	−0.0003 (8)	0.0025 (9)	−0.0016 (9)
C5	0.0154 (11)	0.0124 (11)	0.0231 (12)	−0.0009 (9)	0.0032 (9)	−0.0031 (9)
C6	0.0157 (11)	0.0182 (12)	0.0194 (12)	−0.0018 (9)	0.0051 (9)	−0.0064 (9)
C7	0.0139 (11)	0.0184 (11)	0.0145 (11)	−0.0045 (9)	0.0017 (9)	−0.0009 (9)
C8	0.0134 (11)	0.0151 (11)	0.0206 (12)	0.0008 (9)	0.0011 (9)	0.0004 (9)
C9	0.0281 (13)	0.0266 (12)	0.0179 (12)	−0.0021 (11)	0.0066 (10)	−0.0045 (10)
C10	0.0282 (13)	0.0250 (13)	0.0256 (13)	0.0021 (10)	0.0114 (11)	−0.0080 (10)
C11	0.0218 (13)	0.0177 (12)	0.0330 (14)	0.0033 (10)	0.0145 (11)	0.0045 (10)
C12	0.0152 (11)	0.0158 (11)	0.0333 (13)	0.0008 (9)	0.0082 (10)	0.0069 (10)
C13	0.0171 (12)	0.0225 (13)	0.0515 (17)	0.0025 (10)	0.0110 (12)	0.0025 (12)
C14	0.0108 (12)	0.0218 (13)	0.0561 (18)	0.0002 (10)	−0.0021 (12)	0.0007 (12)
C15	0.0178 (12)	0.0127 (11)	0.0396 (15)	−0.0018 (9)	−0.0046 (11)	0.0041 (10)
C16	0.0166 (12)	0.0138 (11)	0.0299 (13)	−0.0007 (9)	0.0025 (10)	0.0034 (9)
C17	0.0206 (12)	0.0204 (12)	0.0190 (12)	0.0016 (9)	−0.0017 (10)	−0.0012 (9)
C18	0.0147 (11)	0.0201 (11)	0.0254 (13)	0.0015 (9)	0.0007 (10)	0.0010 (10)
C19	0.0141 (11)	0.0180 (12)	0.0180 (12)	−0.0026 (9)	0.0067 (9)	−0.0054 (9)
C20	0.0163 (11)	0.0150 (11)	0.0153 (11)	0.0001 (9)	0.0048 (9)	−0.0024 (9)
C21	0.0218 (12)	0.0179 (11)	0.0196 (12)	−0.0045 (9)	0.0053 (10)	−0.0018 (9)
C22	0.0322 (14)	0.0164 (11)	0.0174 (12)	−0.0021 (10)	0.0053 (10)	0.0016 (9)
C23	0.0231 (12)	0.0185 (12)	0.0140 (11)	0.0044 (9)	0.0010 (9)	−0.0026 (9)
C24	0.0180 (12)	0.0150 (11)	0.0186 (12)	−0.0004 (9)	0.0040 (10)	−0.0037 (9)

### Geometric parameters (Å, °)

**Table:**

Zn1—N2	2.1215 (17)	C3—C4	1.459 (3)
Zn1—N4	2.1292 (18)	C3—H3	0.9500
Zn1—N6	2.1468 (17)	C4—C5	1.387 (3)
Zn1—N5	2.2239 (18)	C5—C6	1.381 (3)
Zn1—N3	2.2711 (17)	C5—H5	0.9500
Zn1—N7	2.3000 (17)	C6—C7	1.387 (3)
O1—C7	1.342 (3)	C6—H6	0.9500
O1—H1O	0.82 (3)	C7—C8	1.399 (3)
O2—C15	1.341 (3)	C8—H8	0.9500
O2—H2O	0.78 (3)	C9—C10	1.518 (3)
O3—C23	1.344 (3)	C9—H9A	0.9900
O3—H3O	0.80 (3)	C9—H9B	0.9900
O4—N8	1.262 (2)	C10—H10A	0.9900
O5—N8	1.230 (2)	C10—H10B	0.9900
O6—N8	1.258 (3)	C11—C12	1.464 (3)
O7—N9	1.269 (2)	C11—H11	0.9500
O8—N9	1.238 (2)	C12—C13	1.390 (3)
O9—N9	1.230 (2)	C13—C14	1.374 (4)
N1—C17	1.454 (3)	C13—H13	0.9500
N1—C1	1.456 (3)	C14—C15	1.383 (3)
N1—C9	1.460 (3)	C14—H14	0.9500
N2—C3	1.273 (3)	C15—C16	1.401 (3)
N2—C2	1.467 (3)	C16—H16	0.9500
N3—C8	1.328 (3)	C17—C18	1.525 (3)
N3—C4	1.357 (3)	C17—H17A	0.9900
N4—C11	1.273 (3)	C17—H17B	0.9900
N4—C10	1.466 (3)	C18—H18A	0.9900
N5—C16	1.334 (3)	C18—H18B	0.9900
N5—C12	1.350 (3)	C19—C20	1.463 (3)
N6—C19	1.270 (3)	C19—H19	0.9500
N6—C18	1.466 (3)	C20—C21	1.388 (3)
N7—C24	1.331 (3)	C21—C22	1.383 (3)
N7—C20	1.352 (3)	C21—H21	0.9500
C1—C2	1.523 (3)	C22—C23	1.385 (3)
C1—H1A	0.9900	C22—H22	0.9500
C1—H1B	0.9900	C23—C24	1.403 (3)
C2—H2A	0.9900	C24—H24	0.9500
C2—H2B	0.9900		
			
N2—Zn1—N4	105.92 (7)	C7—C6—H6	120.9
N2—Zn1—N6	100.61 (6)	O1—C7—C6	124.66 (19)
N4—Zn1—N6	102.29 (7)	O1—C7—C8	116.43 (19)
N2—Zn1—N5	98.59 (6)	C6—C7—C8	118.88 (19)
N4—Zn1—N5	76.15 (7)	N3—C8—C7	122.95 (19)
N6—Zn1—N5	160.36 (6)	N3—C8—H8	118.5
N2—Zn1—N3	75.58 (6)	C7—C8—H8	118.5
N4—Zn1—N3	166.90 (7)	N1—C9—C10	110.56 (18)
N6—Zn1—N3	90.07 (6)	N1—C9—H9A	109.5
N5—Zn1—N3	90.75 (6)	C10—C9—H9A	109.5
N2—Zn1—N7	161.58 (6)	N1—C9—H9B	109.5
N4—Zn1—N7	92.50 (6)	C10—C9—H9B	109.5
N6—Zn1—N7	75.22 (6)	H9A—C9—H9B	108.1
N5—Zn1—N7	85.25 (6)	N4—C10—C9	109.67 (18)
N3—Zn1—N7	86.40 (6)	N4—C10—H10A	109.7
C7—O1—H1O	114 (2)	C9—C10—H10A	109.7
C15—O2—H2O	115 (2)	N4—C10—H10B	109.7
C23—O3—H3O	113 (2)	C9—C10—H10B	109.7
C17—N1—C1	115.53 (17)	H10A—C10—H10B	108.2
C17—N1—C9	115.15 (17)	N4—C11—C12	119.8 (2)
C1—N1—C9	114.74 (18)	N4—C11—H11	120.1
C3—N2—C2	119.29 (17)	C12—C11—H11	120.1
C3—N2—Zn1	116.76 (14)	N5—C12—C13	121.7 (2)
C2—N2—Zn1	123.53 (13)	N5—C12—C11	115.69 (19)
C8—N3—C4	118.10 (18)	C13—C12—C11	122.6 (2)
C8—N3—Zn1	130.39 (14)	C14—C13—C12	119.5 (2)
C4—N3—Zn1	111.22 (13)	C14—C13—H13	120.2
C11—N4—C10	119.50 (19)	C12—C13—H13	120.2
C11—N4—Zn1	115.64 (16)	C13—C14—C15	119.1 (2)
C10—N4—Zn1	124.77 (14)	C13—C14—H14	120.5
C16—N5—C12	118.85 (19)	C15—C14—H14	120.5
C16—N5—Zn1	128.46 (15)	O2—C15—C14	118.7 (2)
C12—N5—Zn1	111.61 (14)	O2—C15—C16	122.5 (2)
C19—N6—C18	119.28 (18)	C14—C15—C16	118.7 (2)
C19—N6—Zn1	116.48 (14)	N5—C16—C15	122.2 (2)
C18—N6—Zn1	124.15 (13)	N5—C16—H16	118.9
C24—N7—C20	118.41 (18)	C15—C16—H16	118.9
C24—N7—Zn1	130.39 (14)	N1—C17—C18	110.43 (17)
C20—N7—Zn1	110.90 (13)	N1—C17—H17A	109.6
O5—N8—O6	121.8 (2)	C18—C17—H17A	109.6
O5—N8—O4	120.8 (2)	N1—C17—H17B	109.6
O6—N8—O4	117.34 (19)	C18—C17—H17B	109.6
O9—N9—O8	121.13 (18)	H17A—C17—H17B	108.1
O9—N9—O7	118.75 (18)	N6—C18—C17	109.49 (17)
O8—N9—O7	120.05 (17)	N6—C18—H18A	109.8
N1—C1—C2	110.47 (17)	C17—C18—H18A	109.8
N1—C1—H1A	109.6	N6—C18—H18B	109.8
C2—C1—H1A	109.6	C17—C18—H18B	109.8
N1—C1—H1B	109.6	H18A—C18—H18B	108.2
C2—C1—H1B	109.6	N6—C19—C20	121.04 (19)
H1A—C1—H1B	108.1	N6—C19—H19	119.5
N2—C2—C1	108.90 (17)	C20—C19—H19	119.5
N2—C2—H2A	109.9	N7—C20—C21	122.22 (19)
C1—C2—H2A	109.9	N7—C20—C19	116.07 (18)
N2—C2—H2B	109.9	C21—C20—C19	121.70 (19)
C1—C2—H2B	109.9	C22—C21—C20	119.3 (2)
H2A—C2—H2B	108.3	C22—C21—H21	120.3
N2—C3—C4	120.48 (19)	C20—C21—H21	120.3
N2—C3—H3	119.8	C21—C22—C23	118.8 (2)
C4—C3—H3	119.8	C21—C22—H22	120.6
N3—C4—C5	121.93 (19)	C23—C22—H22	120.6
N3—C4—C3	115.58 (18)	O3—C23—C22	119.0 (2)
C5—C4—C3	122.49 (19)	O3—C23—C24	122.3 (2)
C6—C5—C4	119.89 (19)	C22—C23—C24	118.7 (2)
C6—C5—H5	120.1	N7—C24—C23	122.6 (2)
C4—C5—H5	120.1	N7—C24—H24	118.7
C5—C6—C7	118.2 (2)	C23—C24—H24	118.7
C5—C6—H6	120.9		
			
N4—Zn1—N2—C3	−171.27 (15)	Zn1—N2—C2—C1	65.7 (2)
N6—Zn1—N2—C3	82.56 (15)	N1—C1—C2—N2	−54.2 (2)
N5—Zn1—N2—C3	−93.31 (15)	C2—N2—C3—C4	176.38 (18)
N3—Zn1—N2—C3	−4.76 (14)	Zn1—N2—C3—C4	3.6 (2)
N7—Zn1—N2—C3	7.6 (3)	C8—N3—C4—C5	0.0 (3)
N4—Zn1—N2—C2	16.28 (17)	Zn1—N3—C4—C5	174.51 (15)
N6—Zn1—N2—C2	−89.90 (16)	C8—N3—C4—C3	−179.80 (18)
N5—Zn1—N2—C2	94.23 (16)	Zn1—N3—C4—C3	−5.3 (2)
N3—Zn1—N2—C2	−177.21 (16)	N2—C3—C4—N3	1.6 (3)
N7—Zn1—N2—C2	−164.89 (18)	N2—C3—C4—C5	−178.28 (19)
N2—Zn1—N3—C8	178.92 (19)	N3—C4—C5—C6	−0.5 (3)
N4—Zn1—N3—C8	−82.8 (3)	C3—C4—C5—C6	179.28 (19)
N6—Zn1—N3—C8	77.97 (18)	C4—C5—C6—C7	0.2 (3)
N5—Zn1—N3—C8	−82.40 (18)	C5—C6—C7—O1	−177.68 (19)
N7—Zn1—N3—C8	2.79 (18)	C5—C6—C7—C8	0.6 (3)
N2—Zn1—N3—C4	5.33 (13)	C4—N3—C8—C7	0.8 (3)
N4—Zn1—N3—C4	103.7 (3)	Zn1—N3—C8—C7	−172.41 (15)
N6—Zn1—N3—C4	−95.62 (14)	O1—C7—C8—N3	177.27 (18)
N5—Zn1—N3—C4	104.01 (14)	C6—C7—C8—N3	−1.1 (3)
N7—Zn1—N3—C4	−170.80 (14)	C17—N1—C9—C10	−81.2 (2)
N2—Zn1—N4—C11	102.79 (16)	C1—N1—C9—C10	141.00 (19)
N6—Zn1—N4—C11	−152.25 (15)	C11—N4—C10—C9	−123.0 (2)
N5—Zn1—N4—C11	7.64 (15)	Zn1—N4—C10—C9	60.7 (2)
N3—Zn1—N4—C11	8.0 (4)	N1—C9—C10—N4	−55.5 (2)
N7—Zn1—N4—C11	−76.84 (16)	C10—N4—C11—C12	178.35 (19)
N2—Zn1—N4—C10	−80.77 (17)	Zn1—N4—C11—C12	−5.0 (3)
N6—Zn1—N4—C10	24.19 (18)	C16—N5—C12—C13	−0.6 (3)
N5—Zn1—N4—C10	−175.92 (18)	Zn1—N5—C12—C13	−169.65 (17)
N3—Zn1—N4—C10	−175.6 (2)	C16—N5—C12—C11	178.94 (18)
N7—Zn1—N4—C10	99.60 (17)	Zn1—N5—C12—C11	9.8 (2)
N2—Zn1—N5—C16	78.54 (18)	N4—C11—C12—N5	−3.7 (3)
N4—Zn1—N5—C16	−177.07 (18)	N4—C11—C12—C13	175.7 (2)
N6—Zn1—N5—C16	−89.3 (3)	N5—C12—C13—C14	0.2 (3)
N3—Zn1—N5—C16	3.01 (17)	C11—C12—C13—C14	−179.3 (2)
N7—Zn1—N5—C16	−83.31 (17)	C12—C13—C14—C15	0.6 (3)
N2—Zn1—N5—C12	−113.68 (14)	C13—C14—C15—O2	177.5 (2)
N4—Zn1—N5—C12	−9.30 (14)	C13—C14—C15—C16	−0.9 (3)
N6—Zn1—N5—C12	78.5 (2)	C12—N5—C16—C15	0.2 (3)
N3—Zn1—N5—C12	170.79 (14)	Zn1—N5—C16—C15	167.21 (15)
N7—Zn1—N5—C12	84.46 (14)	O2—C15—C16—N5	−177.84 (19)
N2—Zn1—N6—C19	−159.92 (15)	C14—C15—C16—N5	0.5 (3)
N4—Zn1—N6—C19	91.03 (16)	C1—N1—C17—C18	−82.7 (2)
N5—Zn1—N6—C19	7.9 (3)	C9—N1—C17—C18	139.83 (19)
N3—Zn1—N6—C19	−84.58 (15)	C19—N6—C18—C17	−114.6 (2)
N7—Zn1—N6—C19	1.68 (15)	Zn1—N6—C18—C17	61.9 (2)
N2—Zn1—N6—C18	23.52 (16)	N1—C17—C18—N6	−55.2 (2)
N4—Zn1—N6—C18	−85.53 (16)	C18—N6—C19—C20	177.74 (18)
N5—Zn1—N6—C18	−168.71 (18)	Zn1—N6—C19—C20	1.0 (3)
N3—Zn1—N6—C18	98.86 (16)	C24—N7—C20—C21	1.3 (3)
N7—Zn1—N6—C18	−174.88 (17)	Zn1—N7—C20—C21	−173.13 (16)
N2—Zn1—N7—C24	−98.6 (3)	C24—N7—C20—C19	−179.69 (18)
N4—Zn1—N7—C24	80.29 (18)	Zn1—N7—C20—C19	5.9 (2)
N6—Zn1—N7—C24	−177.66 (19)	N6—C19—C20—N7	−5.1 (3)
N5—Zn1—N7—C24	4.42 (18)	N6—C19—C20—C21	174.0 (2)
N3—Zn1—N7—C24	−86.64 (18)	N7—C20—C21—C22	−0.4 (3)
N2—Zn1—N7—C20	74.9 (2)	C19—C20—C21—C22	−179.4 (2)
N4—Zn1—N7—C20	−106.20 (14)	C20—C21—C22—C23	−0.9 (3)
N6—Zn1—N7—C20	−4.15 (13)	C21—C22—C23—O3	−177.15 (19)
N5—Zn1—N7—C20	177.93 (14)	C21—C22—C23—C24	1.3 (3)
N3—Zn1—N7—C20	86.87 (14)	C20—N7—C24—C23	−0.8 (3)
C17—N1—C1—C2	137.91 (19)	Zn1—N7—C24—C23	172.30 (15)
C9—N1—C1—C2	−84.5 (2)	O3—C23—C24—N7	177.91 (19)
C3—N2—C2—C1	−106.5 (2)	C22—C23—C24—N7	−0.5 (3)

### Hydrogen-bond geometry (Å, °)

**Table:**

*D*—H···*A*	*D*—H	H···*A*	*D*···*A*	*D*—H···*A*
O3—H3O···O6	0.80 (3)	1.90 (3)	2.698 (2)	173 (3)
O1—H1O···O7	0.82 (3)	1.80 (3)	2.597 (2)	163 (3)
O2—H2O···O4^i^	0.78 (3)	1.84 (3)	2.593 (3)	162 (3)

Symmetry codes: (i) −*x*+3/2, −*y*+1/2, −*z*+1.
